# Influence of age and aerobic fitness on the multifractal characteristics of electrocardiographic RR time-series

**DOI:** 10.3389/fphys.2013.00100

**Published:** 2013-05-13

**Authors:** Michael J. Lewis, Melitta A. McNarry

**Affiliations:** Applied Sports, Technology, Exercise and Medicine Research Group, College of Engineering, Swansea UniversitySwansea, UK

**Keywords:** heart rate, multifractal, self-similarity, exercise, ageing, fitness, ECG

## Abstract

Multifractal properties of electrocardiographic inter-beat (RR) time-series offer insight into its long-term correlation structure, independently of RR variability. Here we quantify multifractal characteristics of RR data during 24-h diurnal-nocturnal activity in healthy participants. We tested the hypotheses that (1) age, gender and aerobic fitness influence RR multifractal properties, and that (2) these are influenced by circadian variation. Seventy adults (39 males) aged 19–58 years and of various fitness levels were monitored using 24-h ECG. Participants were dichotomized by median age and fitness for sub-group analysis. Gender and fitness were independent of age (*p* = 0.1, *p* > 0.5). Younger/older group ages were substantially different (*p* < 0.0005) and were independent of gender and fitness. Multifractality was quantified using the probability spectrum of Hölder exponents (h), from which modal h (h^*^) and the full-width and half-widths at half-maximum measures (FWHM, HWHM+, and HWHM−) were derived. FWHM decreased (*p* = 0.004) and h^*^ increased (*p* = 0.011) in older people, indicating diminished long-range RR correlations and weaker anti-persistent behavior. Anti-persistent correlation (h^*^) was strongest in the youngest/fittest individuals and weakest in the oldest/least fit individuals (*p* = 0.015). Long-range correlation (HWHM+/FWHM) was strongest in the fittest males and weakest in the least fit females (*p* = 0.007–0.033). Multifractal RR characteristics in our healthy participants showed strong age-dependence, with diminished long-range anti-persistent correlation in older people. Circadian variation of these characteristics was influenced by fitness and gender: fitter males and females of all ages had the greatest degree of multifractality or long-range order. Multifractal characterization appears to be a useful method for exploring the physiological basis of long-term correlation structure in RR time-series as well as the benefits thereon of physical fitness training.

## Introduction

Linear (descriptive statistical) methods such as time- and frequency-domain heart rate variability (HRV) analysis have been used for the analysis of cardiac inter-beat (RR) interval time-series. The legitimacy of such methods depends upon the validity of certain assumptions, such as the normality of RR sample distribution. Previous studies have shown that reductions in HRV are associated with both advancing age (Ryan et al., 1994; Umetani et al., 1998; Kuo et al., 1999; Voss et al., 2012) and low aerobic fitness (Dixon et al., 1992; Melanson and Freedson, 2001; Gamelin et al., 2007; Albinet et al., 2010). We have also recently shown that, whilst the lower HRV in older individuals is predominantly attributable to “ageing,” a concomitant decline in aerobic fitness has an additional modulating influence on HRV (McNarry and Lewis, 2012). We and others (Furlan et al., 1990; Lombardi et al., 1992; Huikuri et al., 1994; McNarry and Lewis, 2012) have also observed a circadian variation in HRV in healthy and cardiac disease patients, with a general dominance of parasympathetic-mediated HRV during the night and of sympathetically-mediated HRV during the day.

However, many biological signals are irregular, inhomogeneous and highly autocorrelated and thus a more appropriate candidate model for RR data might be a stochastic fractal process with a given autocorrelation function (Lewis et al., 2012). Accordingly, these so-called non-linear properties of the time-series data have been investigated to assess the correlational properties of RR time-series rather than their variability (Galaska et al., 2008). Such techniques include measures of data structure such as self-similarity and fractal or scaling descriptions (de Godoy et al., 2009).

Fractal signals are typically long-memory processes with a slowly decaying autocorrelation function (Fielding, 1992). In the frequency domain, this corresponds to a 1/*f*-like spectral density function, with the lower frequencies having greater power and the slope of a straight line fitted to the log periodogram being defined as the spectral exponent (Schroeder, 1991; Meyer et al., 1998a,b; Meyer and Stiedl, 2003). Signals are said to be monofractal if they are adequately characterized by a single scaling exponent that is stationary over time. However, it has been recognized that monofractal characteristics do not fully describe the physiological dynamics underpinning RR time-series behavior (Meyer et al., 1998a; Goldberger et al., 2002; Meyer and Stiedl, 2003). Moreover, during extended periods of analysis (that usually involve multiple physiological states) we should allow that the scaling behavior of RR time-series data will not be governed by a single parameter but instead by a number of local scaling exponents. Data with this characteristic are termed *multifractal* and are characterized by the histogram of Hölder exponents, *h*, known as the singularity spectrum (Muzy et al., 1991, 1993; Goldberger et al., 2002; Turiel et al., 2006) that generally spans 0 < *h* < 1.5. Values of the spectral *h* < 0.5 correspond to anti-persistent or negatively correlated behavior and values of *h* > 0.5 correspond to persistent or positively correlated behavior.

Multifractal characteristics have been widely observed in RR time-series (Frisch and Parisi, 1985; Halsey et al., 1986; Ivanov et al., 1999; Amaral et al., 2001; Ivanov et al., 2001; Goldberger et al., 2002; Chiu et al., 2007; Wang et al., 2007), these features reflect the combined influence of heart rate regulatory mechanisms that act mutually independently on different time scales and are interconnected across scales by self-similar processes (Meyer and Stiedl, 2003; Galaska et al., 2008). In this paradigm, “healthy” cardiac function is the capacity to adapt to a variety of exogenous or endogenous stimuli and this is reflected by enhanced multifractal properties of the time-series (for example, a wider distribution of *h* values). In contrast, the occurrence of simpler dynamics (e.g., white noise or a purely periodic oscillation) causes degradation of fractal complexity and indicates poor cardiac function or maladaptivity (Ivanov et al., 2001). Furthermore, we have previously observed that there are no significant correlations between multifractal parameters and the commonly used HRV measures during pre- and post-exercise resting conditions (Lewis et al., 2012). We have also noted from principal component analysis that the correlation structure between multifractal parameters is relatively unperturbed by physical exercise, and is therefore robust to physical state changes. Similarly, Meyer and Stiedl (2003) observed that postural (standing, supine) changes and physiological (respiratory frequency) changes did not influence the RR multifractal spectrum. These authors also observed an absence of circadian variation in RR multifractality in either health individuals or heart failure patients. Altered RR multifractality has now been associated with cardiac pathology, ageing, posture and pharmaceutical autonomic mediators (Ivanov et al., 1999, 2001; Amaral et al., 2001; Meyer and Stiedl, 2003; Chiu et al., 2007; Wang et al., 2007; Makowiec et al., 2011), but notably the influence of aerobic fitness on multifractality has not previously been assessed.

In this study we report the multifractal characteristics of 24 h RR time-series data from healthy male and female participants, this sample group having a range of ages and aerobic fitness levels. We sought to test the hypothesis that age, gender, and aerobic fitness each have an appreciable influence on the multifractal properties of cardiac RR data. Furthermore, we wished to examine whether such influences might be subject to circadian variation. This represents an important addition to our previous report on the HRV-ageing-fitness relationship (McNarry and Lewis, 2012) owing to the proven independence of HRV and multifractal measures, indicating that HRV and multifractality reflect different physiological influences. To our knowledge there has been no previous investigation of this type. The aims of this study were therefore (1) to quantify the structural complexity of cardiac RR time-series using multifractal measures over a 24 h period, and (2) to examine the influences of age, gender, aerobic fitness, and circadian variation on these measures.

## Materials and methods

### Ethics statement

Prior to testing, participants were informed of the protocol and possible risks of participation, and gave written consent to participate. All procedures were approved by the local ethics committee (Abertawe Bro Morgannwg University Health Board) and were conducted in accordance with the Declaration of Helsinki. All data were analysed anonymously.

### Participants

Seventy adults (Range [Median] values: age 19.0–57.5 (32.8) years, BMI 17.2–30.0 (23.5) kg·m^−2^; 39 male) volunteered for the study. The participants were all recreationally active, but not highly trained. Participants were asked to arrive at the laboratory in a rested and fully hydrated state, at least 2 h postprandial and to avoid strenuous exercise in the 24 h preceding each testing session. Participants were also asked to refrain from caffeine and alcohol 6 and 24 h before each test, respectively.

### Measurements

Participants first completed a ramp incremental exercise test for determination of the maximal (peak) rate of oxygen uptake ($\dot{V}{\text{O}}_{2}\text{p}$) and the gas exchange threshold (GET). In this test 3 min of baseline cycling was completed at 0W and then the work rate was increased at a rate of 20–30 W·min^−1^ until the limit of tolerance. An electronically braked cycle ergometer (Lode Excalibur, Groningen, Netherlands) was used and participants were asked to maintain a cadence of 70–80 rpm. Breath-by-breath pulmonary gas-exchange data were collected continuously during the incremental tests and averaged over consecutive 5-s periods (Oxycon Pro, Jaeger, Germany). The $\dot{V}{\text{O}}_{\text{2peak}}$ was taken as the highest 10-s average value attained before the subject's volitional exhaustion in the test. The GET was determined by the V-slope method (Beaver et al., 1986).

After a period of at least 1 week, participants returned to the laboratory and a Reynolds Lifecard CF digital Holter recorder (Spacelabs Medical Ltd., Hertford, UK) was attached to them to record a three-lead ECG continuously for 24 h. The ECG leads were positioned in the modified V5, CC5, modified V5R electrode configuration. This system provided ECG data with a sample accuracy of 2.5 μV (magnitude of least significant bit; 12-bit resolution) and 1024 Hz sampling frequency. ECG recordings were analysed using a Reynolds Pathfinder digital analyser (Spacelabs Medical Ltd., UK). Beat-to-beat cardiac interval (RR) values were automatically measured and exported for further analysis using the Reynolds Research Tools software (Spacelabs Medical Ltd., UK). All subsequently calculated cardiac variables (heart rate and multifractal properties of RR time-series) were quantified during four contiguous 6-h periods: “Morning” (6.00 am–12.00 pm); “Afternoon” (12.00 pm–6.00 pm); “Evening” (6.00 pm–12.00 am); “Night” (12.00 am–6.00 am).

### Multifractal characterisation of time-series

Multifractals are conceptually a series of concurrent fractal processes occurring across multiple, hierarchical scales that interleave to generate the observed signal. The local scaling behavior in the neighborhood of a singularity is characterized by *h* (Hölder exponent) that represents the degree of signal smoothness: small values of *h* correspond to relatively irregular time-series and large values to more regular (smoother) time-series (Muzy et al., 1991, 1993; Turiel et al., 2006). These scaling exponents can be grouped into one of a number of singularity components, each of which comprises all values with equivalent *h* and has a fractal support with dimension *D*(*h*) (also known as the Hausdorff dimension of the set of exponents of *h*). The plot of *D*(*h*) vs. *h* for all singularity components, also known as the singularity spectrum, represents a complete statistical description of a multifractal process.

Multifractal analysis was performed using the CamBA software (http://www-bmu.psychiatry.cam.ac.uk/software/) that uses algorithms available on Physionet (http://www.physionet.org/) (Goldberger et al., 2000). Singularity spectra were obtained via the wavelet transform modulus maxima (WTMM) method. Full details of this method are given elsewhere (Bacry et al., 1993; Muzy et al., 1993). In brief, the continuous wavelet transform of a time-series was obtained with the third derivative of the Gaussian function as the analysing wavelet (Ivanov et al., 2001). The connected local maxima of the transform represent the partition function *Z*_*q*_(*a*) that describes the information contained in a system at each of its scales *a* (Muzy et al., 1994), which in turn is represented by the sum of its moments. The scaling of the partition function can be expressed as a power law [*Z*_*q*_(*a*) ~ *a*^τ(*q*)^] which for multifractal behavior has an exponent that is non-linear with the moments of the function (Vicsek, 1993; Takayasu, 1997). The fractal dimension *D*(*h*) is a function of the local Hurst exponent (*h*) and it is related to τ(*q*) through the Legendre transformation *D*(*h*) = *qh* − τ(*q*), where *h* = *d*τ/*dq*. A plot of *D(h)* against is *h* is referred to as the multifractal spectrum. For statistical testing, the singularity spectra were parameterized by the modal value, h^*^, the half-widths at half-maximum (HWHM) for the side of the spectrum with *h* < h^*^ (*HWHM*−) and for *h* > h^*^ (*HWHM*+), and the full width at half-maximum (FWHM) of the spectrum (Wink et al., 2008).

### Statistical analysis

Median values were calculated for age, $\dot{V}\text{O}2\text{p}$ and GET and were used to dichotomise participants into Younger-Older, Low-High $\dot{V}\text{O}2\text{p}$ and Low-High GET groups, so that they could be used as grouping variables in subsequent ANOVA models. Following confirmation of Normality, repeated measures ANOVA models with main factors “Time” (levels: Morning, Afternoon, Evening, Night) and “Group” (Age, Gender, Fitness [$\dot{V}{\text{O}}_{2}\text{p}$] and Fitness [GET]) were used to assess the magnitudes of the individual cardiac variables. Two- and three-way interaction effects between the main factors were also assessed. During the interpretation of ANOVA results, Mauchly's test was used to determine the validity of the Sphericity assumption and thus to guide the appropriate consultation of test results. For each ANOVA model, *post-hoc* testing using the Bonferroni adjustment identified pair-wise differences in the analysed variables. Simple linear regression analysis was used to examine the relationships between multifractal variables, age and fitness. Two surrogate data sets were created from the original data to enable assessment of the underlying structural model for the RR data: (1) a sample-shuffled data set was used to test the null hypothesis that RR data obey an independent identically distributed (uncorrelated) model, and (2) data transformed using amplitude adjusted Fourier transformation (AAFT; Kugiumtzis, 2000) to test the null hypothesis that RR data conform to a linear stochastic process that has undergone a non-linear transformation. All analyses were conducted using the PASW Statistics package version 18 (SPSS, Chicago, IL). Statistical significance was accepted as *P* < 0.05. Effect sizes were quantified as partial eta squared (η^2^). All data presented in the text represent Mean ± *SD*. Error bars in the figures represent the SEM (standard error in the mean).

## Results

**Figure 1:**
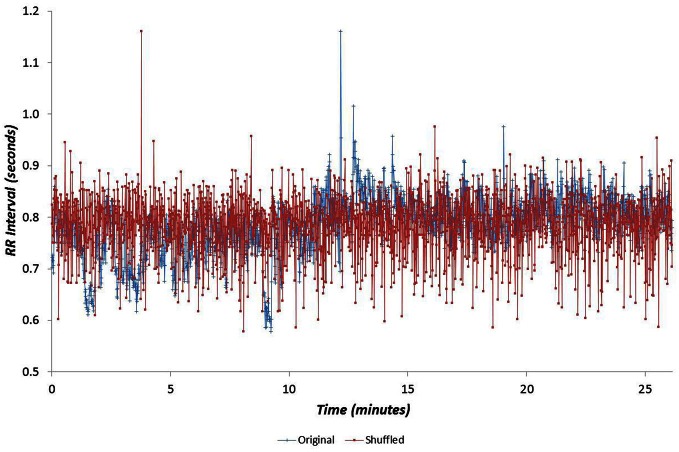
**Example cardiac interval (RR) time-series for the Morning period, shown with the same data following data shuffling**.

**Figure 2:**
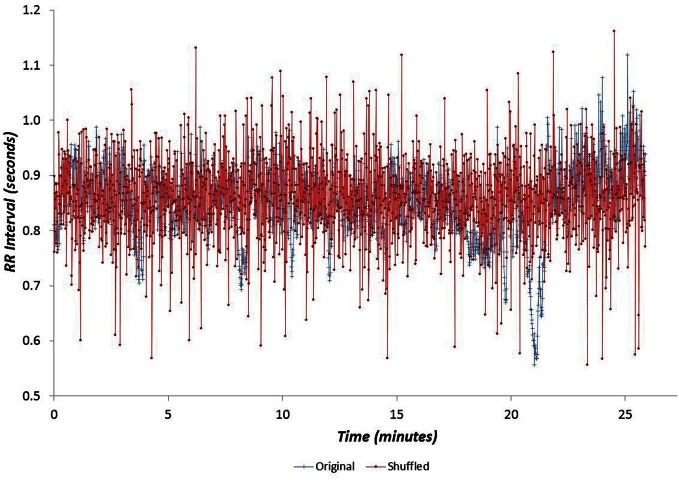
**Example cardiac interval (RR) time-series for the Night period, shown with the same data following data shuffling**.

**Figure 3:**
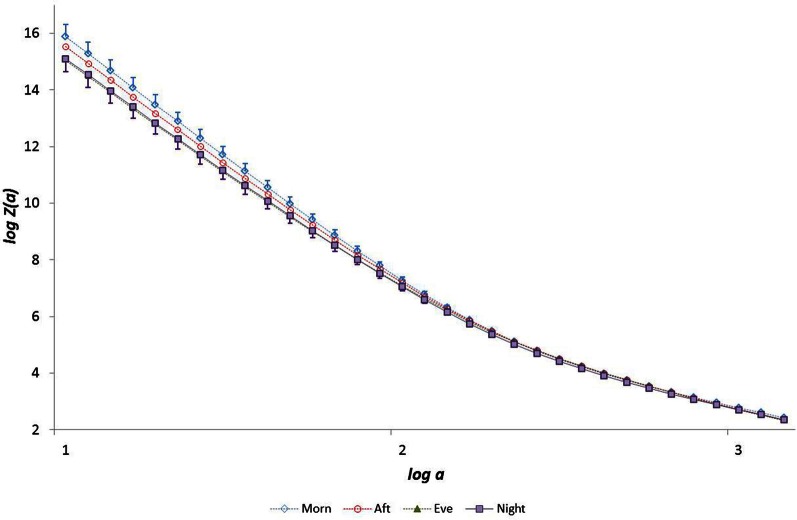
**The partition function *Z*(*a*) from the WTMM procedure as a function of scale *a* (for moment *q* = −5)**.

Data for all seventy participants were analysed. The median values for age, $\dot{V}{\text{O}}_{2}\text{p}$ and GET were 32.8 years, 3.1 l min^−1^ and 1.7 l min^−1^ respectively. The age and fitness distributions for the dichotomized participants groups are shown in Table 1. Younger and older groups did not differ in terms of fitness (expressed as either peak $\dot{V}{\text{O}}_{2}$ and GET) but males were fitter than females (*p* < 0.0005). High and low fitness groups did not differ in terms of age. Figures 1 and 2 show examples of typical RR data sets for the Morning and Night periods, together with the same data following data shuffling. The partition function from the WTMM procedure is shown as a function of scale in Figure 3 for each day-night period. Table 2 presents the statistical results of group and temporal trend comparisons for HR and for the multifractal characteristics of RR time-series.

**Table 1:** **Age and “fitness” distributions for the dichotomised groups (gender, age, $\dot{V}{\text{O}}_{2}\text{p}$, GET)**.

**Group**	***n***	**Age (years)**	***p***	**$\dot{V}O_{2}p$ (l min^−1^)**	***p***	**GET (l min^−1^)**	***p***
All	70	34.4 ± 12.4		3.08 ± 1.00		1.62 ± 0.58	
Male	39	37.2 ± 13.1	0.092	3.23 ± 0.94	<0.0005	1.72 ± 0.50	<0.0005
Female	31	32.1 ± 11.4		2.86 ± 1.05		1.48 ± 0.68	
Younger	35	23.9 ± 4.1	<0.0005	3.06 ± 1.04	0.738	1.63 ± 0.65f	0.555
Older	35	44.9 ± 8.2		3.09 ± 0.97		1.61 ± 0.53	
Low GET	35	35.3 ± 13.1	0.463	2.86 ± 0.92	<0.0005	1.47 ± 0.58	<0.0005
High GET	35	33.5 ± 11.7		3.27 ± 1.04		1.76 ± 0.57	
Low $\dot{V}{\text{O}}_{2}\text{p}$	35	35.4 ± 13.3	0.541	2.86 ± 0.96	<0.0005	1.49 ± 0.60	<0.0005
High $\dot{V}{\text{O}}_{2}\text{p}$	35	33.3 ± 11.4		3.28 ± 1.00		1.74 ± 0.56	

Values are mean ± SD. $\dot{V}O_{2}p$, peak oxygen uptake; GET, gas exchange threshold.

**Table 2:** **Heart rate and multifractal singularity spectrum parameters as a function of “time of day”: (A) for the whole group and for (B) “gender,” (C) “age” and (D,E) “fitness” sub-groups**.

**Sample group**	**Period**	**HR (bpm)**	h^*^	**HWHM−**	**HWHM+**	**FWHM**
**(A)**						
All	Morning	69.5 ± 1.3	0.31 ± 0.01^#^	0.22 ± 0.01	0.52 ± 0.02	0.74 ± 0.02
	Afternoon	73.4 ± 1.5	0.28 ± 0.01	0.22 ± 0.01	0.53 ± 0.02	0.75 ± 0.02
	Evening	71.3 ± 1.6	0.27 ± 0.01	0.21 ± 0.01	0.54 ± 0.02	0.74 ± 0.03
	Night	59.3 ± 1.1^#^	0.28 ± 0.01	0.19 ± 0.01	0.43 ± 0.03	0.63 ± 0.03
Time effect		*p* < 0.0005	*p* < 0.0005	ns	ns	ns
**(B)**						
Male	Morning	65.2 ± 1.5	0.29 ± 0.01	0.21 ± 0.01	0.54 ± 0.03	0.75 ± 0.04
	Afternoon	71.4 ± 2.0	0.27 ± 0.01	0.21 ± 0.01	0.55 ± 0.03	0.76 ± 0.03
	Evening	68.8 ± 2.2	0.27 ± 0.01	0.21 ± 0.01	0.56 ± 0.04	0.77 ± 0.04
	Night	56.7 ± 1.6	0.28 ± 0.01	0.20 ± 0.01	0.43 ± 0.04	0.63 ± 0.04
Female	Morning	74.9 ± 1.8	0.32 ± 0.02	0.23 ± 0.02	0.49 ± 0.03	0.71 ± 0.03
	Afternoon	76.1 ± 2.2	0.29 ± 0.01	0.22 ± 0.02	0.51 ± 0.03	0.73 ± 0.03
	Evening	74.4 ± 2.2	0.28 ± 0.01	0.20 ± 0.01	0.51 ± 0.03	0.71 ± 0.03
	Night	62.6 ± 1.5	0.29 ± 0.01	0.19 ± 0.01	0.43 ± 0.03	0.62 ± 0.03
Group effect		ns	ns	ns	ns	ns
Group interaction effect		ns	ns	ns	(Gender × $\dot{V}{\text{O}}_{2}\text{p}$) *p* = 0.007	(Gender × $\dot{V}{\text{O}}_{2}\text{p}$) *p* = 0.033
Group(s) × Time effect		ns	ns	(Gender × Age × *t*) *p* = 0.025	ns	Ns
**(C)**						
Younger	Morning	69.9 ± 1.9	0.29 ± 0.01	0.21 ± 0.01	0.54 ± 0.03	0.75 ± 0.03
	Afternoon	77.3 ± 2.0	0.27 ± 0.01	0.22 ± 0.01	0.57 ± 0.03	0.78 ± 0.04
	Evening	75.5 ± 2.1	0.26 ± 0.01	0.19 ± 0.01	0.62 ± 0.03	0.81 ± 0.03
	Night	61.0 ± 1.8	0.26 ± 0.01	0.18 ± 0.01	0.46 ± 0.04	0.63 ± 0.04
Older	Morning	69.1 ± 1.8	0.32 ± 0.02	0.22 ± 0.02	0.50 ± 0.03	0.72 ± 0.03
	Afternoon	69.6 ± 2.1	0.29 ± 0.01	0.21 ± 0.02	0.50 ± 0.03	0.71 ± 0.03
	Evening	67.0 ± 2.2	0.29 ± 0.01	0.22 ± 0.01	0.46 ± 0.03	0.67 ± 0.04
	Night	57.6 ± 1.4	0.31 ± 0.01	0.21 ± 0.01	0.41 ± 0.03	0.62 ± 0.04
Group effect		0.002	0.004	ns	0.011	0.048
Group interaction effect		ns	(Age × $\dot{V}{\text{O}}_{2}\text{p}$) *p* = 0.015	ns	ns	ns
Group(s) × Time effect		(Age × t) *p* < 0.0002	(Age × GET × *t*) *p* < 0.0005	(Age × GET × *t*) *p* < 0.0005	ns	ns
			(Age × $\dot{V}{\text{O}}_{2}\text{p}$ × *t*) *p* = 0.001			
**(D)**						
Low GET	Morning	73.3 ± 1.9	0.30 ± 0.01	0.21 ± 0.01	0.50 ± 0.03	0.71 ± 0.03
	Afternoon	76.6 ± 2.2	0.28 ± 0.01	0.22 ± 0.02	0.53 ± 0.03	0.75 ± 0.03
	Evening	74.3 ± 2.1	0.27 ± 0.01	0.20 ± 0.01	0.54 ± 0.03	0.74 ± 0.03
	Night	62.4 ± 1.2	0.28 ± 0.01	0.19 ± 0.01	0.47 ± 0.03	0.66 ± 0.03
High GET	Morning	65.8 ± 1.6	0.31 ± 0.02	0.22 ± 0.02	0.53 ± 0.03	0.76 ± 0.03
	Afternoon	70.3 ± 1.9	0.28 ± 0.01	0.21 ± 0.01	0.54 ± 0.04	0.75 ± 0.04
	Evening	68.2 ± 2.3	0.28 ± 0.01	0.21 ± 0.01	0.54 ± 0.04	0.74 ± 0.04
	Night	56.2 ± 1.8	0.28 ± 0.01	0.20 ± 0.01	0.34 ± 0.04	0.59 ± 0.04
Group effect		ns	ns	ns	ns	ns
Group × Time effect		ns	(GET × *t*) *p* = 0.013	(GET × *t*) *p* = 0.003	ns	ns
**(E)**						
Low $\dot{V}\text{O}2\text{p}$	Morning	73.8 ± 2.0	0.31 ± 0.02	0.22 ± 0.01	0.51 ± 0.03	0.73 ± 0.03
	Afternoon	74.5 ± 2.4	0.28 ± 0.01	0.21 ± 0.01	0.52 ± 0.03	0.73 ± 0.03
	Evening	73.6 ± 2.4	0.27 ± 0.01	0.20 ± 0.01	0.54 ± 0.03	0.74 ± 0.03
	Night	61.7 ± 1.4	0.28 ± 0.01	0.18 ± 0.01	0.47 ± 0.03	0.65 ± 0.04
High $\dot{V}{\text{O}}_{2}\text{p}$	Morning	65.5 ± 1.4	0.30 ± 0.01	0.21 ± 0.01	0.53 ± 0.03	0.74 ± 0.03
	Afternoon	72.4 ± 1.8	0.28 ± 0.01	0.22 ± 0.01	0.55 ± 0.03	0.77 ± 0.04
	Evening	69.1 ± 2.1	0.28 ± 0.01	0.21 ± 0.01	0.53 ± 0.04	0.75 ± 0.04
	Night	57.1 ± 1.7	0.28 ± 0.01	0.20 ± 0.01	0.40 ± 0.04	0.60 ± 0.04
Group effect		ns	ns	ns	ns	ns
Group × Time effect		($\dot{V}{\text{O}}_{2}\text{p}$ × *t*) *p* = 0.010	($\dot{V}{\text{O}}_{2}\text{p}$ × *t*) *p* < 0.0005	($\dot{V}{\text{O}}_{2}\text{p}$ × *t*) *p* = 0.029	ns	ns

# Significant difference compared to all other periods, p < 0.0005.

Values are mean ± SD. $\dot{V}O_{2}$, oxygen uptake; GET, gas exchange threshold; HR, heart rate; h, Hölder exponent; HWHM-, half width at half-maximum (HWHM) for the side of the spectrum with h < h^*^; HWHM+, half width at half-maximum (HWHM) for the side of the spectrum with h > h^*^; FWHM, full width at half-maximum (FWHM) of the spectrum.

**Figure 4:**
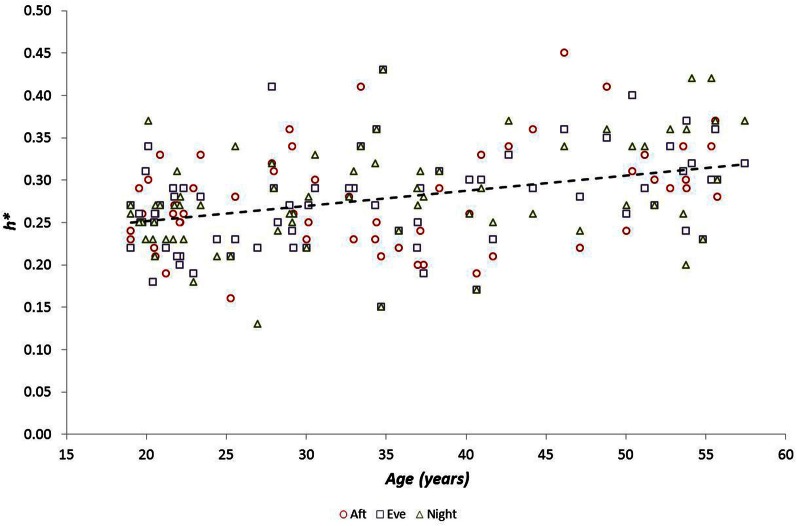
Modal h (h^*^) as a function of participant age during Afternoon, Evening and Night periods (there was no relationship between h^*^ and age during the Morning period).

**Figure 5:**
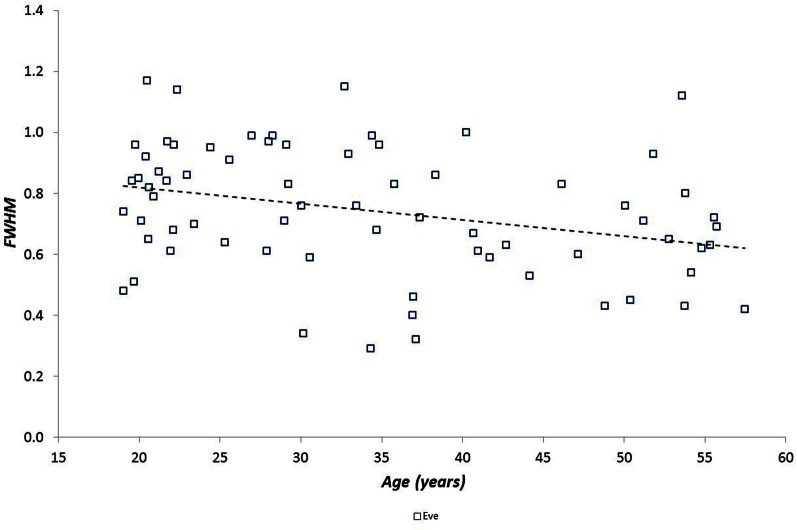
Spectral width (FWHM) as a function of participant age during the Evening period (there was no relationship between h^*^ and age for the other periods).

**Figure 6:**
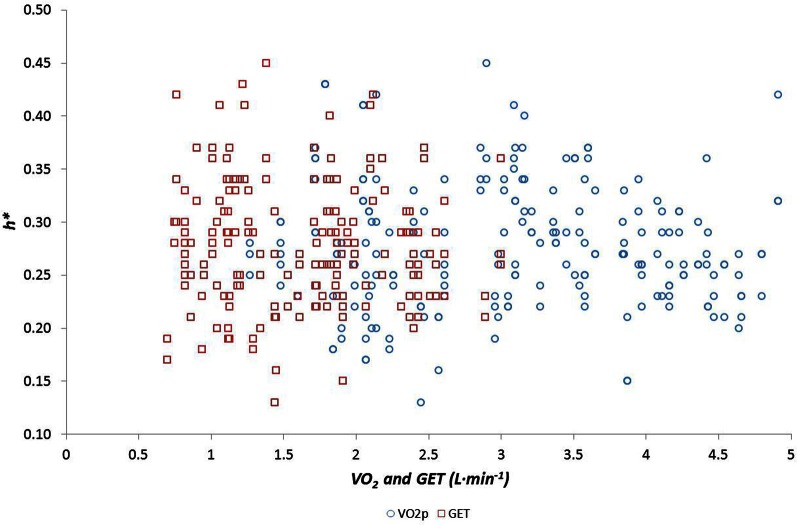
Modal h (h^*^) as a function of fitness, measured as both the gas exchange threshold (GET) and the maximal rate of oxygen uptake ($\dot{V}{\text{O}}_{2}\text{p}$).

We examined the relationship between h^*^ and FWHM using simple linear regression, showing that there was no relationship between these variables (*r* = −0.034, *p* = 0.58). We also examined the relationships between multifractal properties (h^*^ and FWHM) and both age and fitness using simple linear regression. Age was related to h^*^ during the Afternoon (*r* = 0.33, *p* = 0.006), Evening (*r* = 0.38, *p* = 0.001) and Night periods (*r* = 0.41, *p* = 0.0004) (Figure 4) but was weaker during the Morning (*r* = 0.22; *p* = 0.0620), and age was related to FWHM during the Evening period (*r* = −0.385 and −0.313, *p* = 0.001 and 0.008 respectively) (Figure 5). There were no significant relationships between h^*^/FWHM and fitness ($\dot{V}{\text{O}}_{2}\text{p}$ or GET) (Figure 6).

**Figure 7:**
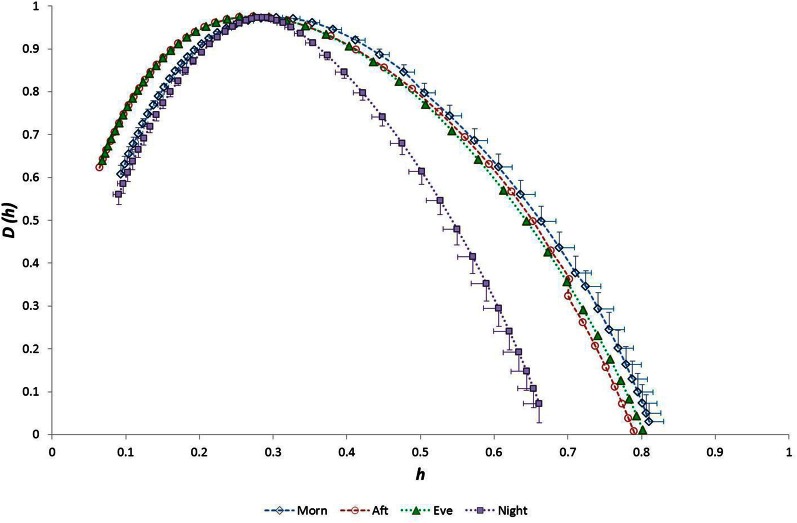
**Singularity multifractal spectra by “time of day” for the whole group**.

**Figure 8:**
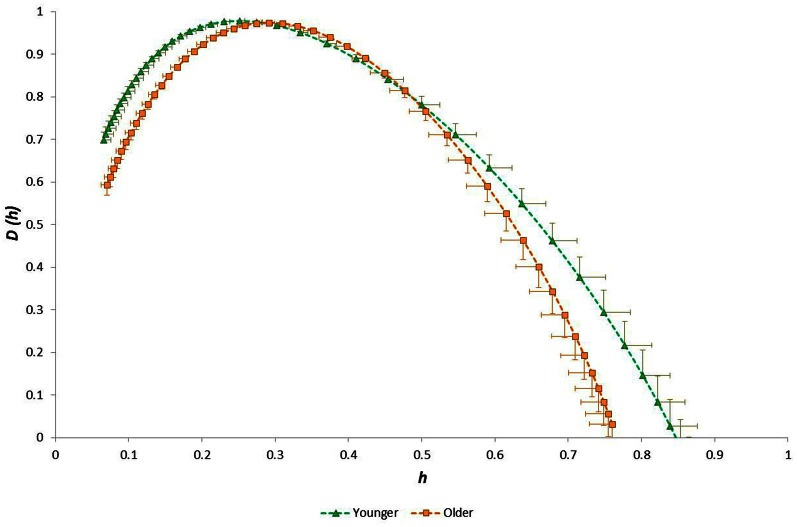
**Singularity multifractal spectra for younger and older groups (shown for the Evening period; other time periods showed similar results)**.

**Figure 9:**
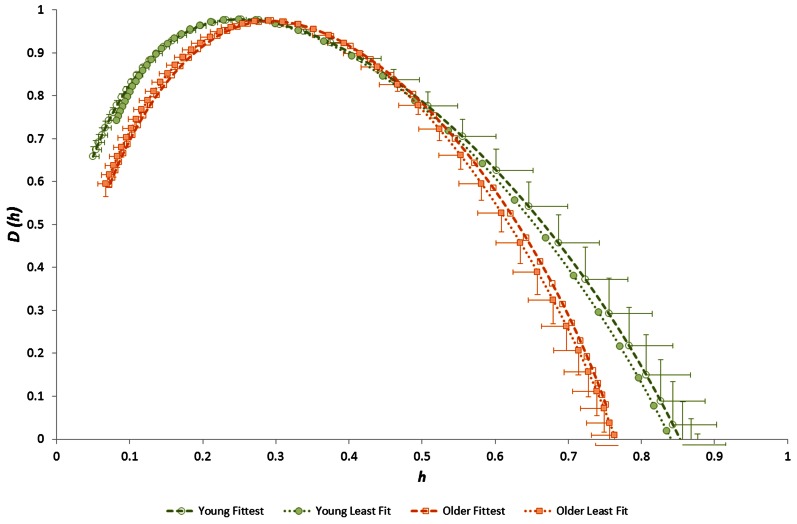
**Singularity multifractal spectra for younger and older groups after separation by fitness level (shown for the Evening period; other time periods showed similar results)**.

Considering the “time of day” influence on cardiac variables in the whole group, HR was lowest at night (*p* < 0.0005, η^2^ = 0.17) and h^*^ was greatest in the morning (*p* < 0.0005, η^2^ = 0.15). Although qualitatively it appeared that HWHM+ and FWHM were diminished at night (Figure 7), statistical testing showed this to be marginal and not significant (*p* = 0.054−0.062). With regard to the sub-groups, neither gender nor fitness level influenced any of the cardiac variables. However, age had a significant influence on HR (*p* = 0.002, η^2^ = 0.16) and on the multifractal spectral variables: h^*^ (*p* = 0.004, η^2^ = 0.13), HWHM+ (*p* = 0.011, η^2^ = 0.11) and FWHM (*p* = 0.048, η^2^ = 0.06) (Figure 8). There was an Age × $\dot{V}{\text{O}}_{2}\text{p}$ interaction influence on h^*^ (*p* = 0.015, η^2^ = 0.10) (Figure 9). Whilst there was also a Gender × $\dot{V}{\text{O}}_{2}\text{p}$ interaction influence on HWHM+ (*p* = 0.007, η^2^ = 0.12) and FWHM (*p* = 0.033, η^2^ = 0.08), we note that the vast majority of females (27 out of 31) were in the lower $\dot{V}{\text{O}}_{2}\text{p}$ group, which perhaps biased this result.

The 24-h variation in HR was influenced by Age (*p* = 0.002; η^2^ = 0.08) and $\dot{V}{\text{O}}_{2}\text{p}$ (*p* = 0.010; η^2^ = 0.06). The 24-h variation in h^*^ was influenced by GET (*p* = 0.013; η^2^ = 0.06), $\dot{V}{\text{O}}_{2}\text{p}$ (*p* < 0.0005; η^2^ = 0.12), Age × GET (*p* < 0.0005; η^2^ = 0.14) and Age × $\dot{V}{\text{O}}_{2}\text{p}$ (*p* = 0.001; η^2^ = 0.09), and the 24-h variation in HWHM- was influenced by GET (*p* = 0.003; η^2^ = 0.08), $\dot{V}{\text{O}}_{2}\text{p}$ (*p* = 0.029; η^2^ = 0.05), Age × Gender (*p* = 0.025; η^2^ = 0.05) and Age × GET (*p* < 0.0005; η^2^ = 0.12).

**Figure 10:**
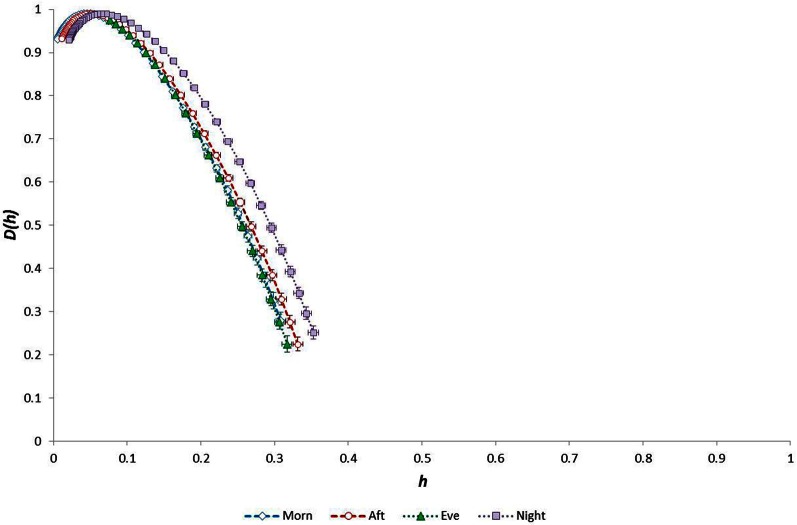
**Singularity multifractal spectra by “time of day” calculated for surrogate data (order-shuffled transformation of original data)**.

**Figure 11:**
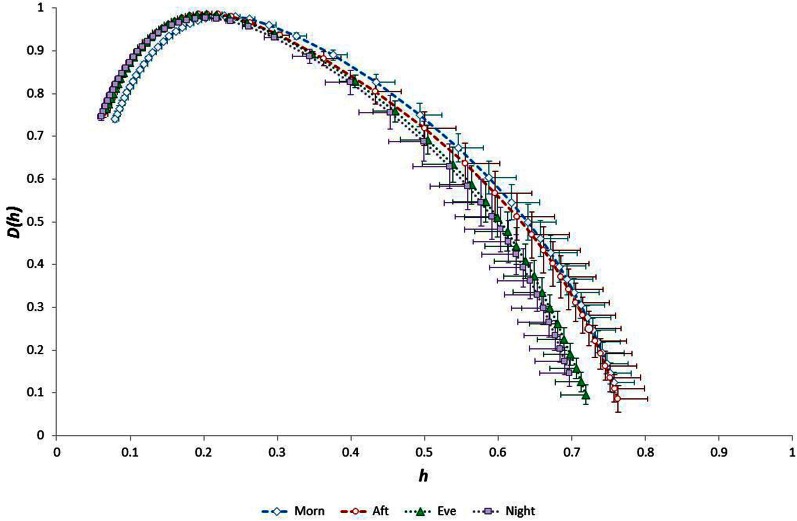
**Singularity multifractal spectra by “time of day” calculated for surrogate data (amplitude adjusted Fourier transformation of original data)**.

Order-shuffled RR data (i.e., with correlation structure removed) shifted the spectra toward to significantly lower h^*^ values (*p* < 10^−38^) and reduced their widths (*p* < 10^−16^) as expected (Figure 10). The spectra retained a form indicative of multifractals after shuffling, i.e., they had appreciable width. The h^*^ value of the shuffled data was significantly greater for Night compared with Morning periods (*p* < 0.0005; η^2^ = 0.25) but spectral width FWHM was equivalent across the four time periods (*p* = 0.11; η^2^ = 0.08). Linear regression analysis showed that the relationships observed between age, h^*^ and FWHM in the original data were all absent from the shuffled data. Compared with the original data, the AAFT surrogate data exhibited slightly reduced h^*^ values (reflecting a whitening of the data, as expected with this method) but their spectral widths were unchanged (Figure 11). As with the original data, h^*^ was greatest in the morning (*p* < 0.0005; η^2^ = 0.23) and spectral width was equivalent across the four time periods (range for FWHM: *p* = 0.17−0.54; η^2^ = 0.01−0.07). The linear relationship between age and h^*^ observed in the original data were maintained in the AAFT surrogate data during the Morning (*r* = 0.39, *p* = 0.001), Afternoon (*r* = 0.36, *p* = 0.003) and Evening (*r* = 0.33, *p* = 0.005) periods but was weaker during the Night (*r* = 0.19, *p* = 0.110). The 24-h mean values for h^*^ and FWHM for the original, order-shuffled surrogate and AAFT surrogate data were: h^*^ = 0.055 ± 0.001 vs. 0.284 ± 0.004 vs. 0.218 ± 0.004, and FWHM = 0.318 ± 0.005 vs. 0.713 ± 0.012 vs. 0.675 ± 0.018).

## Discussion

We observed that the multifractal properties of cardiac RR time series in our sample group of healthy 19–58 year olds were independent of both gender and fitness level, but they were substantially influenced by age. Specifically, the modal Hölder exponent value (h^*^) was greater and both HWHM+ and FWHM were smaller in the older group (Figure 8). This age-related narrowing of the multifractal spectrum as h^*^ increased reflects a reduction in long-range correlations and a weaker anti-persistent correlation relationship in the RR time-series of older people. Our observation of diminished multifractality is consistent with the concept of a less “complex” physiological control system (with fewer or weaker physiological/autonomic control components) for HR in older people.

Multifractality in time series can be attributed to either a broad probability density function of the fluctuations (difference between time-points) of the time series or non-linear features related to different long-range correlations for small and large fluctuations (Kantelhardt et al., 2002) or a mixture of the two. We used two types of surrogate data to examine the likely models underlying the structure of our RR data. First, we tested the null hypothesis that the RR data obey an independent identically distributed (uncorrelated) model by using a shuffled (order randomized) version of the original data. Significant differences in the multifractal spectra (h^*^ and width) for the original and shuffled data led to the rejection of this model (as expected) for all day-night periods. The spectra retained a form indicative of multifractals (i.e., they had appreciable width). We next used surrogate data created from the original data using AAFT. This allowed us to test the null hypothesis that RR data conform to a linear stochastic process that has undergone a non-linear transformation. Multifractal spectral properties were largely retained in this surrogate data: there was evidence of some whitening of the data (reduction in h^*^) but spectral width was unchanged. In this case we do not reject the null hypothesis. In combination our results suggest that a non-linearly transformed linear stochastic process with wide probability density function is an appropriate candidate model for our data.

Notably, Ivanov et al. (1999) observed reductions in long-range correlation and a diminution of anti-persistent correlation in RR time-series following both sympathetic and parasympathetic pharmaceutical blockade in young individuals. A dominant multifractal influence from the parasympathetic component of the autonomic nervous system was also evident in that study. We suggest that the diminished anti-persistent correlation of heart rate observed in older people in the present study can be interpreted as a reflection of their reduced autonomic (parasympathetic) responsiveness to systemic physiological changes.

We also noted a significant combined (interaction) effect of age and aerobic fitness ($\dot{V}{\text{O}}_{2}\text{p}$) on h^*^ (Figure 9), indicating that anti-persistent correlation in RR time-series is strongest in the youngest/fittest individuals and weakest in the oldest/least fit individuals. This is a further indication that anti-persistent long-term correlation behavior characterizes the healthiest/fittest state of cardiac control. There was also a combined effect of gender and fitness ($\dot{V}{\text{O}}_{2}\text{p}$) on HWHM+ and FWHM, indicating that long-range correlation is strongest in the fittest males and weakest in the least fit females.

As expected from numerous previous observations, HR was lowest at night and was lower in older and less fit individuals. Considering the multifractal properties of RR time-series data, we observed that the weakest anti-persistent correlation behavior in these data occurred during the morning, and there was a trend toward reduced long-range correlation during the night (Figure 7). We also noted that (1) age and fitness ($\dot{V}{\text{O}}_{2}\text{p}$) influenced the 24-h temporal trend in HR, (2) fitness and the interaction between age-fitness affected the 24-h trend in h^*^ (the strength of anti-correlated behavior), and (3) fitness and the interactions between both age-gender and age-fitness GET affected the 24-h trend in HWHM- (the degree of multifractality).

It therefore appears that there is circadian variation in the multifractal properties of RR time-series, and moreover this temporal is intricately determined by a person's age, gender and fitness level. This contrasts with the previously reported absence of circadian variation in RR multifractality (Meyer and Stiedl, 2003) but concurs with recent evidence of circadian variation of multifractality in the very low frequency bandwidth of RR (Makowiec et al., 2011). These differing observations might be related to the ranges of age and fitness in the sample groups in each study. We may only tentatively suggest here that there is practical relevance in this circadian dependence of multifractality on age and fitness. That said, it would seem logical to surmise that an individual with “abnormal” 24-h RR multifractal behavior might have a physiologically compromised heart rate control mechanism.

Our results suggest that the magnitude and type of long-range correlation in RR time-series and their circadian variation, as represented by multifractal parameters, are intricately determined by a person's age and fitness level but not their gender. It was beyond the scope of this work to examine the practical or clinical relevance of these results. However, this work shows that there is a need for clearly defined “normal” sub-group reference values for RR multifractal parameters. Since fitness is modifiable, in future studies we will seek to examine whether it is also possible to “train” individuals of relatively low “fitness” using physical exercise to re-align their multifractal characteristics to peer-group normative values.

We were also not able to verify that participants were asleep throughout the night period, and we acknowledge that we do not know whether sleep-wake transitions might have influenced our results. It might be possible in future studies to use polysomnography to assess for this potential confounder. We must also comment that there are alternative methods for calculating the multifractality spectra, and some authors might argue against the WTMM method that we employed. For example Turiel et al. (2006) observed that this method causes linearization and possible corruption of the right-hand tail of the multifractal spectrum. However, these authors also noted that the method also has some advantages, such as filtration of spurious and oscillating components in the analysed data. Furthermore, we have used this method in previous work (Lewis et al., 2012) and we are satisfied that it is robust in the analysis of heart rate time-series data.

In conclusion, the multifractal characteristics of RR time-series in our group of healthy participants displayed clear evidence of a strong age-dependence (long-range anti-persistent correlation being diminished in older people), whilst the 24-h circadian variations of these characteristics were influenced by fitness and gender. We observed that fitter individuals of all ages had the greatest degree of multifractality or long-range order. Assuming that multifractal RR parameters reflect the performance of cardiac autonomic control, our study suggests that multifractality is a contender surrogate measure of the cardiac impact of autonomic ally-mediated factors. Such modifiable extrinsic factors include pharmaceutical and exercise interventions, the latter suggesting a physical “training” process. Multifractal characterization therefore appears to be a useful method for exploring the physiological basis of long-term correlation structure in RR time-series as well as the benefits thereon of physical fitness training.

### Conflict of interest statement

The authors declare that the research was conducted in the absence of any commercial or financial relationships that could be construed as a potential conflict of interest.
